# A novel exploration of treating local skin infections around totally implantable venous access ports: port repositioning technique vs. port re-implantation technique

**DOI:** 10.1186/s40001-025-03039-8

**Published:** 2025-08-13

**Authors:** Qiulian Sun, Ling Tang, Jiefei Cheng, Dongqing Ren, Xinchun Guo

**Affiliations:** 1 Department of Radiology, The Fifth People’s Hospital of Suzhou, The Affiliated Infectious Diseases Hospital of Soochow University, No.10, Guangqian Road, Suzhou, 215100 Jiangsu Province China; 2 Department of Radiology, Taizhou Fourth People’s Hospital, No.99, Gulou Road, Tai Zhou City, 225300 Jiangsu Province China; 3 Department of Radiology, The Fifth People’s Hospital of Taizhou, Tai Zhou City, 225300 Jiangsu Province China; 4 Department of Interventional Radiology, Jiangyin Hospital Affiliated to Nantong University, No. 3, Yingrui Road, Jiang Yin City, 214400 China; 5 Department of Interventional Radiology, Jiangyin Hospital Affiliated to Nantong University, No. 3, Yingrui Road, Jiang Yin City, 214400 Jiangsu Province China; 6 Yangtze University, Jingzhou City, 434020 Hubei Province China

**Keywords:** Totally implantable venous access port, Periport infection, Port repositioning technique, Re-implantation of the port

## Abstract

**Objective:**

To conduct a comparative assessment of the safety and efficacy of the port repositioning technique and the port re-implantation technique in treating periport skin infections associated with totally implantable venous access ports (TIVAPs).

**Methods:**

A retrospective analysis was performed on 35 patients who presented with periport skin infections at Jiangyin People's Hospital between June 2016 and August 2022. Among them, 15 patients in Group A underwent port repositioning surgery, while 20 patients in Group B received port re-implantation surgery. Clinical data of all patients were meticulously collected, including postoperative wound healing status and the functionality of the repositioned or re-implanted TIVAPs.

**Results:**

In Group A, the median age was 58 years (IQR 46–63 years); in Group B, the median age was 60.5 years (IQR 54.3–70 years). The median BMI of Group A patients was 22.2 kg/m^2^ (IQR 20.4–23.5 kg/m^2^); the median BMI of Group B patients was 21.5 kg/m^2^ (IQR 20.5–23.1 kg/m^2^). Technical success was achieved in all patients (100%), and all ports were functional postoperatively. In Group A, the median indwelling time of the repositioned TIVAPs was 7 months (IQR 4–11 months); in Group B, the median indwelling time of the newly re-implanted TIVAPs was 5 months (IQR 3–8 months); there was no statistically significant difference between the two groups (P > 0.05).

**Conclusion:**

Compared with the port re-implantation technique, the port repositioning technique for periport skin infections is a minimally invasive and cost-effective approach. Nevertheless, further investigations with a larger number of cases are required to comprehensively validate its safety and reliability.

**Supplementary Information:**

The online version contains supplementary material available at 10.1186/s40001-025-03039-8.

## Introduction

Totally implantable venous access port (TIVAP), commonly referred to as the venous access port, plays a crucial role in the standard treatment of the majority of tumors. Chemotherapy delivery via an TIVAP serves to mitigate the venous toxicity associated with chemotherapeutic agents and alleviate the anxiety and discomfort stemming from repeated intravenous injections [1,2]. Nevertheless, the utilization of TIVAP is not devoid of risks. The principal complications associated with it encompass infection, catheter thrombosis, catheter occlusion, catheter fracture, and catheter displacement [3]. Infection stands out as the most prevalent and serious complication of TIVAP, with an incidence rate ranging from approximately 5 to 8% [4–6]. In fact, among the reasons leading to TIVAP removal, infection accounts for 46.2% [7]. Venous access port infections can be broadly categorized into two types: local infections of the subcutaneous pocket or catheter-related bloodstream infections (CRBSIs) [8]. Research has indicated [9] that local infections are not amenable to systemic antibiotic treatment. Instead, the access port along with the surrounding necrotic tissue should be removed using aseptic procedures. However, certain scholars argue [10,11] that the clinical decision to remove the access port should take into account multiple factors. These include the severity of the infection symptoms, the availability of alternative options for the patient's venous access, and the anticipated difficulty in establishing a new access.

**Fig. 1:**
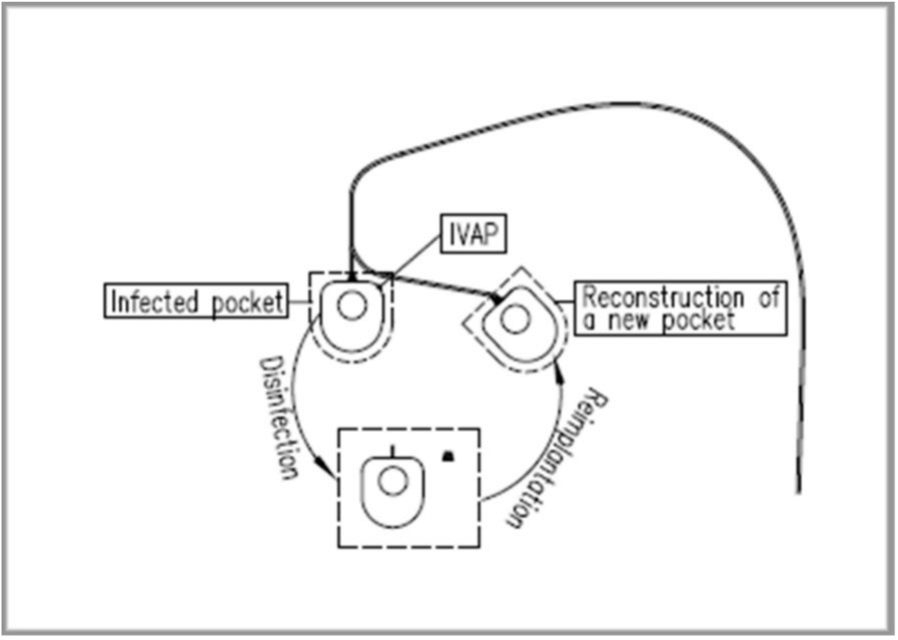
For periport skin infection of a chest wall-implanted port that failed to respond to standard antibiotic therapy, the port was removed via a longitudinal incision along its edge under local anesthesia. After disinfection by soaking in povidone-iodine for 15 min, a new pocket was created 2–3 cm medial to the original site, and the sterilized port was reassembled and re-implanted into the new pocket

While surgical removal of the TIVAP can effectively manage the infection, for patients who still require long-term intravascular access for chemotherapy, a new TIVAP must be implanted, which inevitably involves both surgical risks and considerable financial burden. The port repositioning technique entails creating a new pocket 2–3 cm medial to the original access port under aseptic conditions (Fig. 1). Subsequently, the thoroughly disinfected access port body and its components are reassembled and re-implanted subcutaneously. This study aims to compare the safety and efficacy of the port repositioning technique and the port re-implantation technique in the treatment of local skin infections around the venous access port. The findings are presented below.

## Materials and methods

### Study subjects

A retrospective analysis was carried out on the clinical data (including age, gender, underlying diseases, implantation site of the TIVAP, indwelling duration, indications for port repositioning, complications, etc.) and corresponding imaging records of 35 patients who developed periport skin infections at Jiangyin People's Hospital between June 2016 and August 2022. Specifically, 15 patients in Group A underwent the port repositioning technique, while 20 patients in Group B received port re-implantation technique. The infusion ports used were high-pressure implantable venous ports manufactured by B. Braun (Celsite^®^) and Bard.

Local periport infection was defined as the occurrence of erythema and pain in the skin surrounding the TIVAP and/or the presence of purulent discharge from the port pocket, in the context of negative blood cultures [12]. Indications for port repositioning and re-implantation: Once patients exhibited manifestations of local periport infection, they were administered antibiotics (piperacillin-tazobactam, 4.5 g via intravenous drip every 8 h). After a 7 day treatment course, if there was no marked improvement in the periport infection, they met the criteria for the respective procedures. All patients provided informed consent for the surgical interventions.

### Surgical methods

**Fig. 2.**
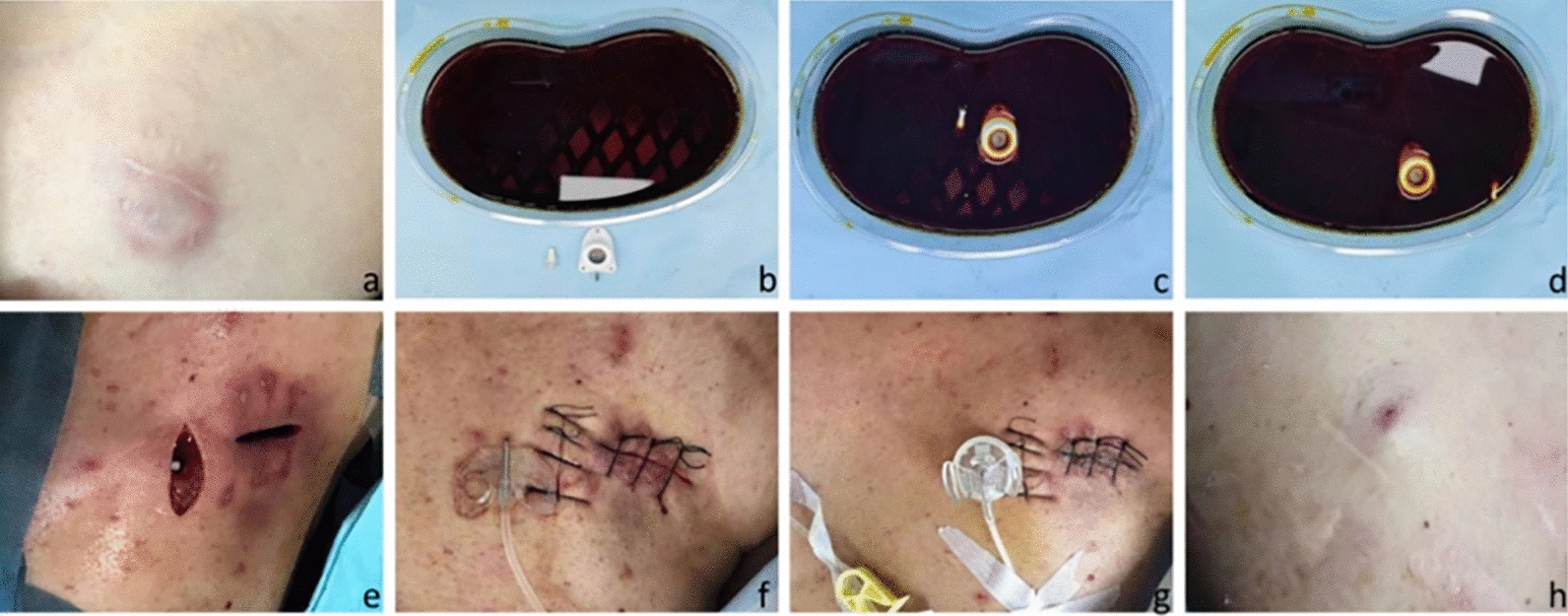
**a** Localized skin infection surrounding the port body on the right upper chest wall showed no remarkable improvement following antibiotic therapy. **b**–**d** Once the port body was extracted, it was immersed in povidone-iodine solution for 15 min to undergo disinfection. **e** A fresh subcutaneous pocket was fashioned, and the disinfected port body, after being reassembled, was re-implanted into this newly created pocket. **f** Post-operatively, chemotherapy was initiated utilizing the re- positioned port. **g** The original and the newly formed subcutaneous pockets began to heal gradually. **h** Fourteen days after the surgery, both the original and the new pockets had achieved satisfactory healing

**Fig. 3:**
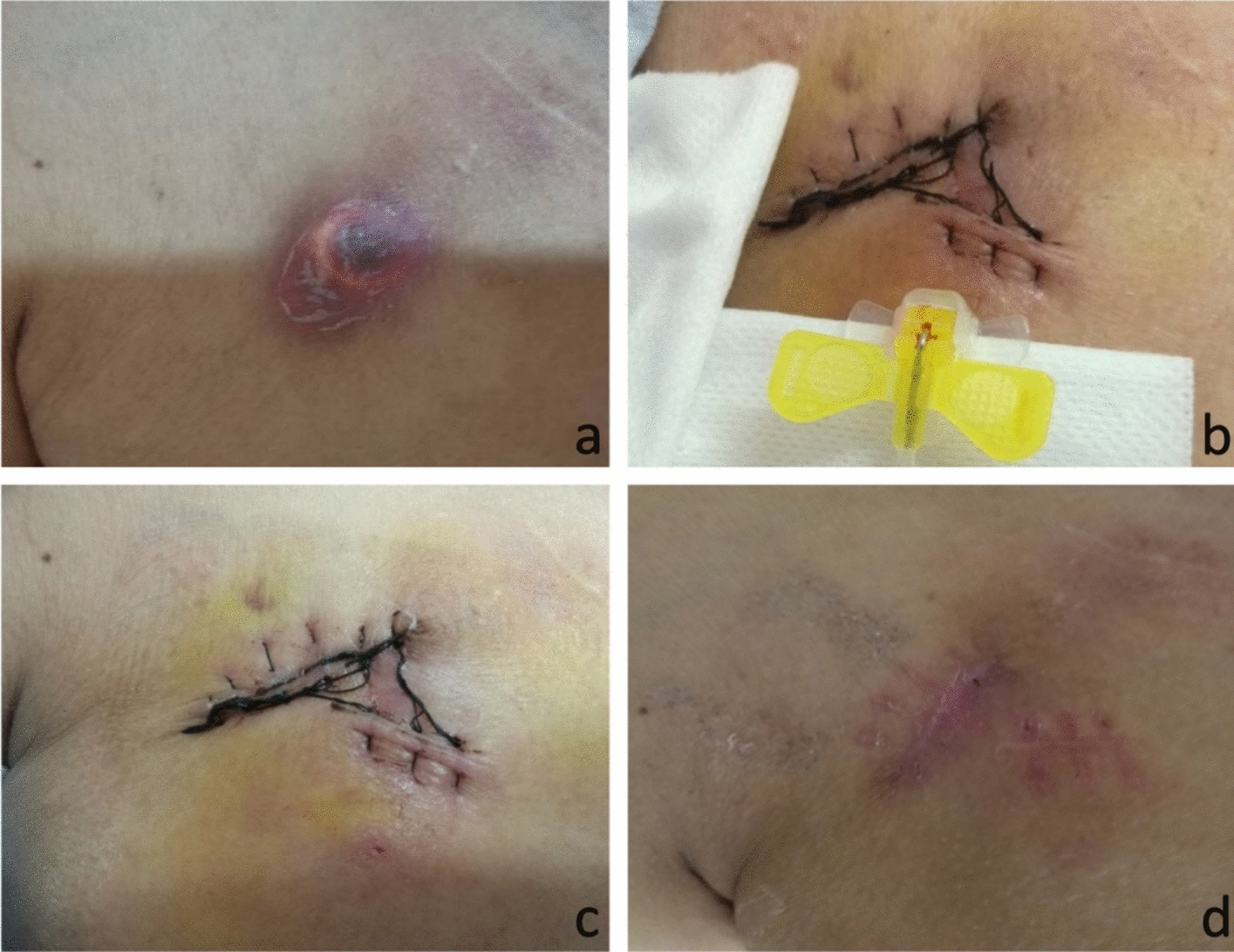
**a**. Local skin infection around the port body on the right upper chest wall, with no significant improvement after antibiotic treatment. **b** A new pocket was created on the left upper chest wall, and a new port was inserted. **c** The operation was completed, and the surgical area on the chest wall was bandaged and fixed. **d** After the operation, the surgical wound on the right upper chest wall healed well, the new pocket on the left upper chest wall also healed well, and the new port was functioning normally

Port Repositioning (Fig. 2, 3): The surgical procedure of port repositioning was carried out by interventionalists in the interventional operating suite. The patient was positioned supine on the DSA examination couch, with the head turned contralaterally to expose the neck and the anterior chest region of the operative field. Following standard disinfection and draping, a longitudinal incision was meticulously made along the periphery of the port body under local anesthesia (Fig. 2e). Subsequently, the port body and its locking mechanism were carefully extracted, and the catheter was clamped, with an external gauze acting as a protective barrier. The port body and locking mechanism were then immersed in a povidone—iodine solution for 15 min, after which they were thoroughly rinsed and set aside. Meanwhile, the catheter at the port's connection site was subjected to comprehensive disinfection (Fig. 2b–d). All necrotic tissue was meticulously excised, and purulent discharge was sent for bacterial culture and antibiotic susceptibility testing, and the surgical site was repeatedly irrigated with a diluted povidone-iodine solution followed by normal saline. The subcutaneous tissue and skin were then sutured in a precise manner. A new subcutaneous pocket was created approximately 2–3 cm medial to the original one (Fig. 2e). Under the guidance of DSA fluoroscopy, the tip of the catheter was carefully adjusted to a position between the fifth (T5) and seventh (T7) thoracic vertebrae. The disinfected port body and its associated components were then reassembled and reimplanted subcutaneously. A puncture needle was used to access the port body, and the aspiration of blood was confirmed to be unobstructed. The port was then sealed with heparinized saline. The incision was sutured, and local pressure dressing and fixation were applied to ensure stability. Upon successful completion of the operation, the patient was safely transferred back to the ward.

**Fig. 4:**
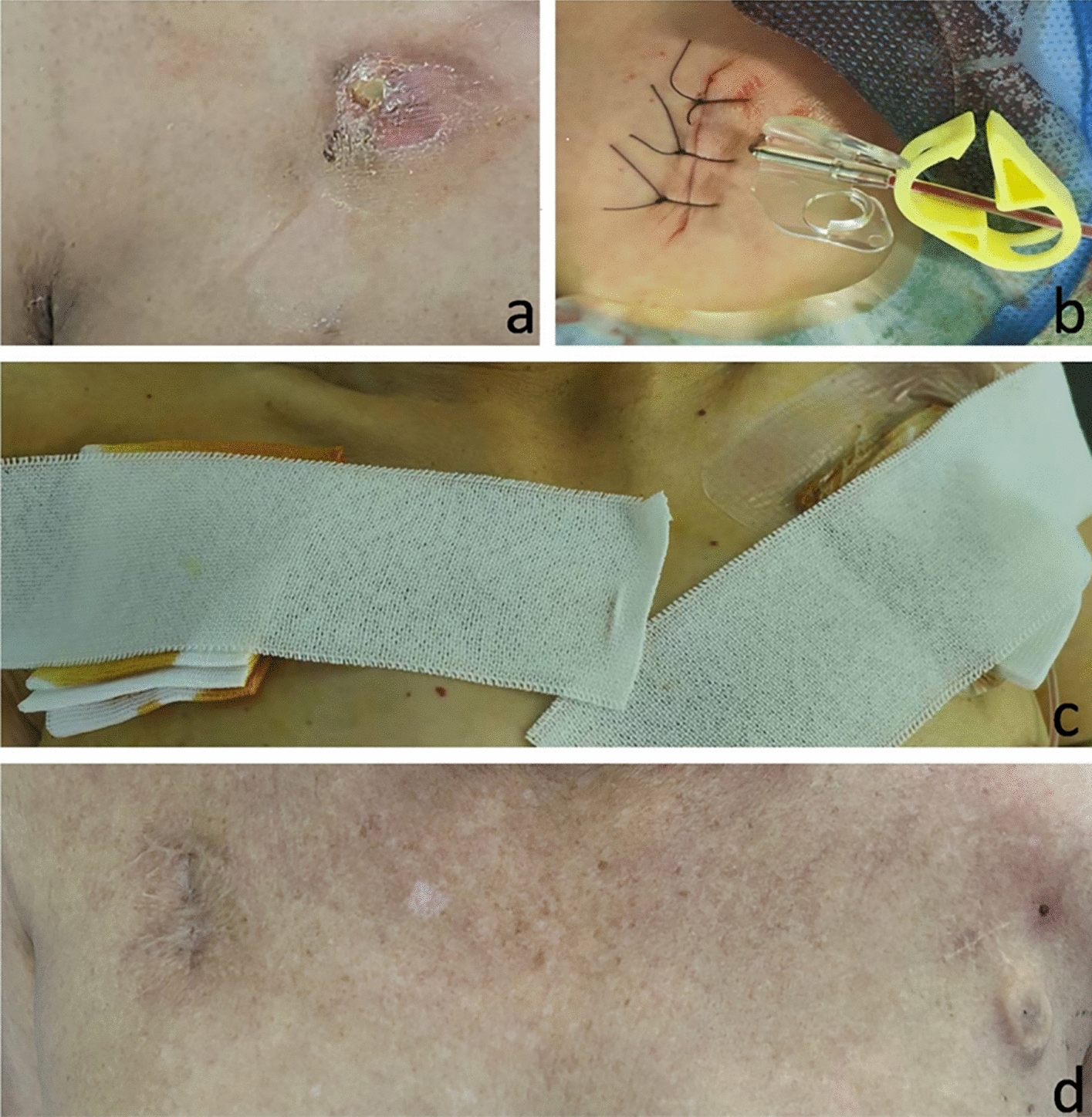
**a** Local skin infection around the port body on the right upper chest wall, showing no significant improvement after antibiotic treatment. **b**, **c** Two days after the operation, chemotherapy was started using the re-positioned port. **d** Fourteen days after the operation, both the original pocket and the new pocket had healed well

Re-implantation of the TIVAP (Fig. 4): The patient was positioned supine on the DSA examination table, with the head turned contralaterally to expose the neck and bilateral anterior chest regions. Inspection of the skin overlying the original port site revealed erythema, exudate, and ulceration (Fig. 4a). DSA fluoroscopy was performed to confirm the normal position and configuration of the port body and catheter. A 2.5-cm long skin incision was made along the ulcerated skin. The superficial fascia was incised, and the fibrous capsule was bluntly dissected to completely remove the catheter and the port body. Local hemostasis was achieved, and all necrotic tissue was thoroughly excised, and purulent material was sent for bacterial culture and drug sensitivity testing. The cystic cavity was then irrigated sequentially with diluted povidone—iodine solution and normal saline. The skin and subcutaneous tissue were closed with interrupted sutures. After local disinfection, a pressure dressing was applied, and the procedure was successfully completed. Following standard aseptic preparation and draping with a fenestrated surgical towel, local anesthesia was infiltrated into an area 3 cm inferior to the mid-point of the clavicle on the contralateral anterior chest wall. An approximately 3-cm transverse incision was made, and the subcutaneous tissue was gently dissected in a blunt manner to create a subcutaneous pocket measuring approximately 2.5 cm × 3 cm. Using the midpoint of the incision as the puncture site, the third segment of the axillary vein was accessed under ultrasound guidance. Once dark-red venous blood was aspirated, a guide wire was advanced into the superior vena cava. Fluoroscopy was used to precisely confirm the correct position of the guide wire. A 7F vascular sheath was then introduced over the guide wire, and the catheter was threaded through the sheath. Under DSA fluoroscopy guidance, the catheter tip was carefully adjusted to a position between the fifth and seventh thoracic vertebrae (T5-T7). The catheter was trimmed to an appropriate length, and a locking device was used to securely connect the catheter to the port body. The port body was then gently placed into the subcutaneous pocket. A butterfly-shaped non-traumatic puncture needle was used to access the port body, and unobstructed aspiration of blood was confirmed. The port was then flushed and sealed with 20 ml of heparinized saline. The incision was closed with sutures (Fig. 4b), and a pressure dressing was applied for immobilization (Fig. 4c). Throughout the procedure, the patient remained in stable condition with normal vital signs. The operation was successfully completed.

### Statistical analysis

Statistical analyses were performed using SPSS version 22.0. Measurement data were expressed as medians with interquartile ranges (IQR). Group comparisons for continuous data were performed using the Mann–Whitney U test. Categorical variables were compared using Fisher’s exact test. Statistical significance was defined as p < 0.05.

## Results

In Group A, where patients underwent port repositioning surgery, the cohort comprised 10 males and 5 females, aged 33 to 76 years, with a median age of 58 years (IQR 46–63 years); the median BMI was 22.2 kg/m^2^ (IQR 20.4–23.5 kg/m^2^). Regarding the underlying primary diseases, there were 2 cases of right-lung adenocarcinoma, 1 case of left-breast invasive ductal carcinoma accompanied by multiple metastases, 1 case of right-breast invasive ductal carcinoma, 1 case of left-lung squamous cell carcinoma, 1 case of left-lung adenocarcinoma, 2 cases of multiple myeloma, 1 case of gastric adenocarcinoma, 2 cases of sigmoid colon cancer, 1 case of myelodysplastic syndrome, 1 case of colon cancer, 1 case of non- Hodgkin lymphoma, and 1 case of pancreatic malignancy. 14 ports were implanted in the right chest wall and 1 in the left chest wall. The port repositioning procedures were successfully carried out for all patients, achieving a technical success rate of 100%. Throughout the surgical procedures, patients reported no significant discomfort, and no severe complications were detected. Postoperatively, patients received intravenous cefoperazone-sulbactam 3.0 g every 12 h for 7 days. For 3 cases, antibiotic therapy was adjusted to biapenem 0.3 g every 8 h on day 5 based on bacterial culture and susceptibility results, continuing for an additional 7 days. Detailed information is provided in Table 1. Two patients developed recurrent local skin infections around the port at 1 month and 3 months post-surgery, respectively.

**Table 1:** Comparison of clinical information between the two groups

Variable	Group A	Group B	P value
Gender (n, %)			P > 0.05
Male	10 (66.7%)	9 (45%)	
Female	5 (33.3%)	11 (55%)	
Age median (IQR) (years)	58 (46, 63)	60.5 (54.3–70)	P > 0.05
BMI median (IQR) (kg/m^2^)	22.2 (20.4–23.5)	21.5 (20.5–23.1)	P > 0.05
Underlying diseases (n, %)			
	Lung adenocarcinoma (3, 20%)	Breast cancer (3, 15%)	
	Breast cancer (2,13.3%)	Gastric adenocarcinoma (3, 15%)	
	Colon cancer (3, 20%)	Colon cancer (5, 25%)	
	Myelodysplastic syndrome (1, 6.7%)	Diffuse large B-cell lymphoma (3,15%)	
	Lung squamous cell carcinoma (1, 6.7%)	Cervical cancer (1, 5%)	
	Multiple myeloma (2, 13.3%)	Lung adenocarcinoma (1, 5%)	
	Gastric adenocarcinoma (1, 6.7%)	Pancreatic cancer (1, 5%)	
	Pancreatic cancer (1, 6.7%)	Intrahepatic cholangiocarcinoma (1, 5%)	
	Non-Hodgkin lymphoma (1, 6.7%)	Non-small cell lung cancer (1, 5%)	
		Gallbladder carcinoma (1, 5%)	
Implantation site of the TIVAP (n, %)			
	Right Chest Wall (14, 93.3%)	Right Chest Wall (20, 100%)	
	Left Chest Wall (1, 6.7%)	Left Chest Wall (20, 100%)	
Duration of surgery Median (IQR) (Minutes)	29 ( 25–35)	44 (40.3–52.8)	P < 0.05
Original port indwelling duration Median (IQR) (Months)	6 ( 4–12)	4.5 ( 3–8.5)	P > 0.05
Port repositioning/re-implantation port indwelling duration Median (IQR) (Months)	7 (4–11)	5 ( 3–8)	P > 0.05
Indications for port repositioning/re-implantation port (n, %)			P > 0.05
	Local infection around the TIVAP port (10, 66.7%)	Local infection around the TIVAP port (14, 70%)	
	Local infection around the TIVAP port with dermal ulceration (4, 26.7%)	Local infection around the TIVAP port with dermal ulceration (4, 20%)	
	Local infection around the TIVAP port with incision dehiscence (1, 6.7%)	Local infection around the TIVAP port with incision dehiscence (2, 10%)	
Complications (n, %)	None	1 (5%)	P > 0.05
The interval from surgery to the first postoperative chemotherapy Median (IQR) (Days)	2 (1–2)	2 (1–2)	P > 0.05
Adjustment of antibiotics (n, %)	(3, 20%)	(2, 10%)	P > 0.05

TIVAP: Totally implantable venous access port

In Group B, patients underwent the procedure of new infusion port implantation. The group consisted of 9 males and 11 females, aged between 38 and 81 years, with a median age of 60.5 years (IQR 54.3–70 years); the median BMI was 21.5 kg/m^2^ (IQR 20.5–23.1 kg/m^2^). The underlying primary diseases were diverse: 3 cases of breast cancer, 3 cases of gastric adenocarcinoma, 2 cases of sigmoid colon cancer, 3 cases of ascending colon cancer, 3 cases of diffuse large B-cell lymphoma, 1 case of cervical cancer, 1 case of adenocarcinoma in the left upper lung lobe, 1 case of pancreatic cancer, 1 case of intrahepatic cholangiocarcinoma, 1 case of right-lung small-cell carcinoma, and 1 case of gallbladder cancer. The initial infusion port was implanted on the right chest wall in 17 patients and on the left chest wall in 3 patients. All new infusion port implantation procedures in Group B were successfully completed, achieving a technical success rate of 100%. During the operations, patients reported no significant discomfort. Post-operatively, one case of pneumothorax was noted, while no severe complications, including hemothorax, arterial injury, arrhythmia, guide-wire kinking, alteration of the puncture site, or wound dehiscence, were detected. Postoperatively, patients were administered intravenous cefoperazone-sulbactam 3.0 g every 12 h for 7 days. In two cases, antibiotic therapy was adjusted to biapenem 0.3 g every 8 h on day 5 based on culture and sensitivity results, with treatment continued for an additional 7 days. Details are presented in Table 1.

There were no statistically significant differences in age, gender and BMI between the two groups (P > 0.05). The median operative time in Group A was 29 min (IQR 25–35 min); the median operative time in Group B was 44 min (IQR 40.3–52.8 min), and the difference in operative time between Groups A and B was statistically significant (P < 0.05). The median indwelling time of the initially implanted TIVAP in Group A was 6 months (IQR 4–12 months); in Group B, the median indwelling time of the initially implanted TIVAP was 4.5 months (IQR 3–8.5 months), and there was no statistically significant difference between the groups (P > 0.05). The median indwelling time of TIVAP after port body repositioning in Group A was 7 months (IQR 4–11 months); the median indwelling time of the newly implanted TIVAP in Group B was 5 months (IQR 3–8 months); there was no statistically significant difference between the two groups (P > 0.05). The median interval between surgery and the first postoperative chemotherapy in Group A was 2 days (IQR 1–2 days); the median interval between surgery and chemotherapy after port reimplantation in Group B was 2 days (IQR 1–2 days); there was no statistically significant difference between the two groups (P > 0.05). There was no statistically significant difference in complications between the two groups (P > 0.05).

## Discussion

In clinical settings, the majority of cancer patients depend on TIVAPs to facilitate antitumor chemotherapy, parenteral nutrition administration, and the management of blood products [13]. Despite the widespread and generally safe use of TIVAP placement, a certain degree of complications remains a concern. Among these, infection stands out as the most critical issue, significantly influencing patient morbidity and mortality rates while also escalating healthcare costs [4]. Findings from relevant research indicate that upon the diagnosis of CRBSIs, the standard treatment protocol commonly encompasses the removal of the device along with systemic anti- infective treatment [14]. In the event of a tunnel or pocket infection, it is typically necessary to promptly remove the catheter. Incision and drainage may be carried out if required, followed by a 7- to 10 day course of antibiotic therapy [10].

A large proportion of cancer patients rely on TIVAPs to support their chemotherapy treatments. Consequently, the removal of an TIVAP can have a detrimental impact on the patients' treatment schedules. The 2016 version of the “Standards of Practice for Intravenous Therapy” clearly states [15] that, when the treatment goals have been achieved, vascular access devices should be replaced as sparingly as possible. This approach is crucial for reducing the occurrence of complications. Furthermore, previous research has shown [16] that TIVAPs are associated with a lower incidence of complications compared to other long-term venous access devices. However, re-implanting a new TIVAP can significantly increase the financial burden on patients and lead to delays in their routine treatment. Therefore, there is an urgent clinical need for more optimal management strategies for local infections around the TIVAP.

In this study, we presented a novel technique for addressing local skin infections around the TIVAP: the port repositioning procedure. We then compared this approach with the method of re-implanting the TIVAP on the contralateral side. Results indicated that there was no statistically significant difference in the duration of infusion port use between the two groups (P > 0.05). There was a statistically significant difference in operative time between the two groups (P < 0.05), with the port body repositioning group having a shorter surgery duration.

The authors believe that the port repositioning procedure presents several distinct advantages when juxtaposed with the approach of removing the existing port and subsequently re-implanting a new one. Although there is no statistically significant difference in the interval between surgery and the first postoperative chemotherapy between the two procedures, patients treated with the port body repositioning technique can commence chemotherapy through the port within 1–2 days postoperatively (Fig. 3b/c), minimizing disruption to their antitumor treatment course, patients undergoing port removal plus new port re-implantation, some have elective contralateral port implantation, which indirectly affects their cancer treatment timeline. Secondly, from a financial perspective, the combined cost of removing an infected TIVAP and then implanting a new one approximates $1240. This breakdown includes the expense of the removal surgery (approximately $155), the cost of the re-implantation surgery (around $155), and the price of a new TIVAP (roughly $930). In stark contrast, the total cost of the port repositioning procedure is confined solely to the surgical fee, which amounts to approximately $200. This significant cost differential translates into a marked reduction in the financial burden borne by patients. Finally, the port body repositioning procedure has a shorter operative time and does not require repeated central venous puncture, thereby eliminating risks associated with new TIVAP implantation, such as puncture-related injuries, air embolism, arrhythmias, pericardial and vascular perforations^[[17]]^.

Several limitations exist in this study: Firstly, as it only addresses chest TIVAP implantations, the findings may not be applicable to port placements at other anatomical locations; Secondly, in the port repositioning group, one patient each developed a recurrence of periport local skin infection at 1 month and 3 months postoperatively. This suggests that there may be inadequate disinfection or debridement in the original infected area, indicating a risk of infection recurrence. Lastly, in the port body repositioning surgery, the port was repositioned 2–3 cm inward, resulting in an unavoidable cranial shift of the catheter tip by approximately 2 cm, potentially increasing risks of catheter displacement, thrombosis, and arrhythmias; However, patients who underwent port body repositioning in this study maintained normal port function postoperatively, with no related complications reported.

Our preliminary clinical practice study indicates that, compared with contralateral port re-implantation, the port body repositioning technique for managing local periport infections is feasible and clinically valuable. However, as this study is a retrospective exploratory investigation with a small sample size, larger prospective studies are needed in the future to evaluate the efficacy and safety of this procedure.

## Supplementary Information

Supplementary Material 1.

## Data Availability

No datasets were generated or analysed during the current study.
