# Supplementary material for: A novel exploration of treating local skin infections around totally implantable venous access ports: port repositioning technique vs. port re-implantation technique
Source: Eur J Med Res. 2025 Aug 13;30:747. doi: 10.1186/s40001-025-03039-8 (PMC12344980; doi:10.1186/s40001-025-03039-8)
Supplement: Supplementary file 1 — Supplementary Material 1. [file 40001_2025_3039_MOESM1_ESM.docx]

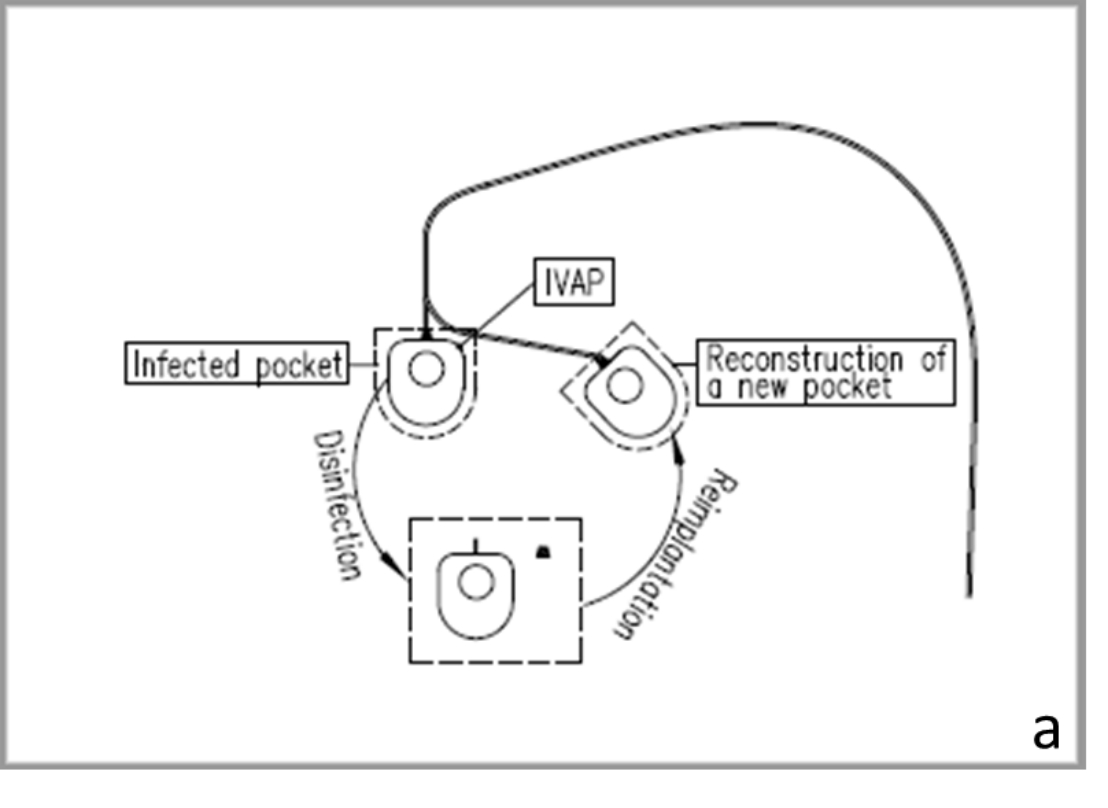


Figure 1 Schematic diagram of the port repositioning surgery.


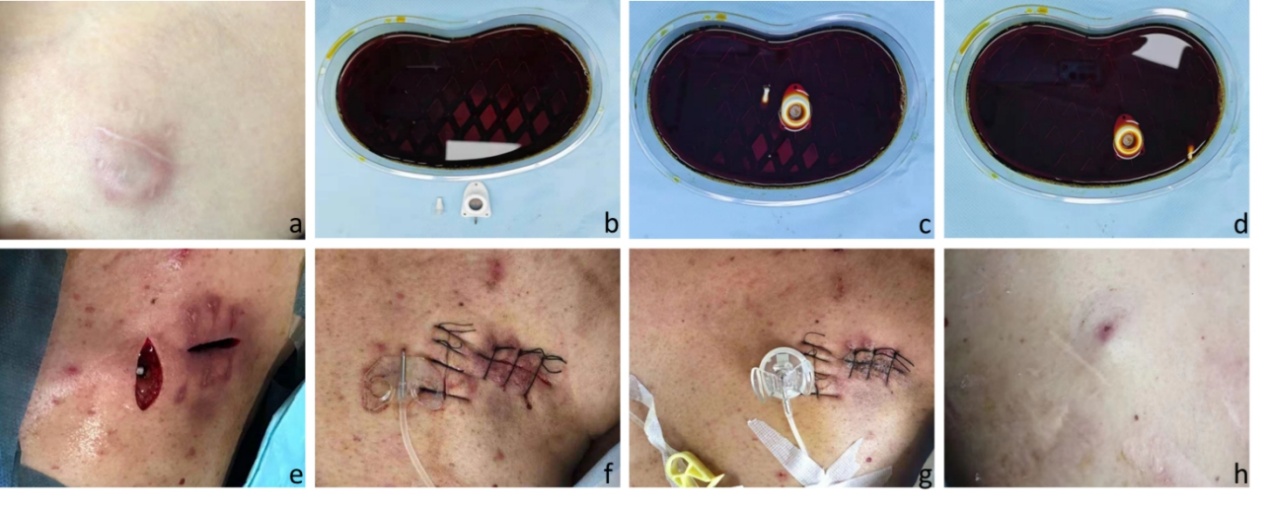


Figure 2. 2a. Localized skin infection surrounding the port body on the right upper chest wall showed no remarkable improvement following antibiotic therapy. 2b-2d. Once the port body was extracted, it was immersed in povidone-iodine solution for 15 minutes to undergo disinfection. 2e. A fresh subcutaneous pocket was fashioned, and the disinfected port body, after being reassembled, was re - implanted into this newly created pocket. 2f. Post-operatively, chemotherapy was initiated utilizing the re- positioned port. 2g. The original and the newly formed subcutaneous pockets began to heal gradually. 2h. Fourteen days after the surgery, both the original and the new pockets had achieved satisfactory healing.


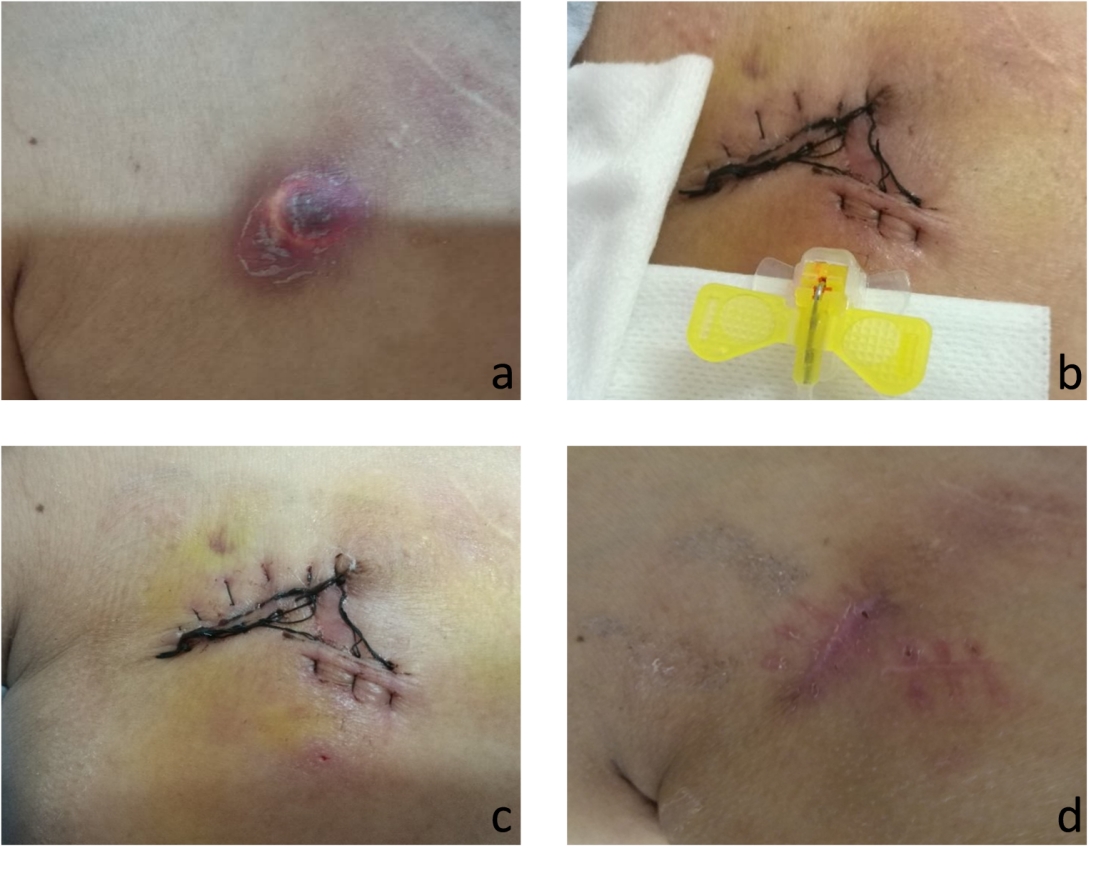


Figure 3. 3a. Local skin infection around the port body on the right upper chest wall, showing no significant improvement after antibiotic treatment. 3b, 3c. Two days after the operation, chemotherapy was started using the re - positioned port. 3d. Fourteen days after the operation, both the original pocket and the new pocket had healed well.


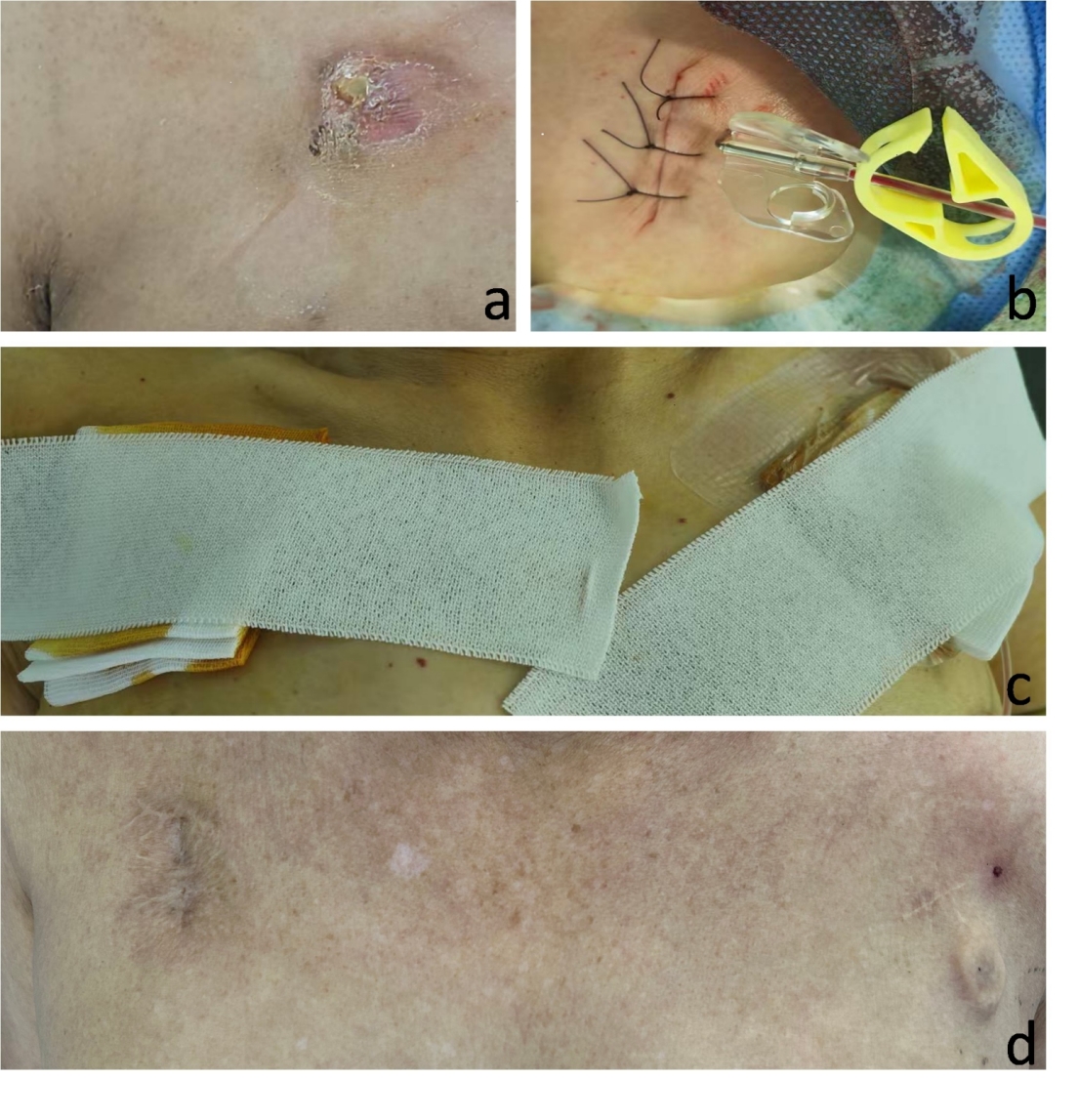


Figure 4. 4a. Local skin infection around the port body on the right upper chest wall, with no significant improvement after antibiotic treatment. 4b. A new pocket was created on the left upper chest wall, and a new port was inserted. 4c. The operation was completed, and the surgical area on the chest wall was bandaged and fixed. 4d. After the operation, the surgical wound on the right upper chest wall healed well, the new pocket on the left upper chest wall also healed well, and the new port was functioning normally.
